# Supplementary material for: Assembly and glycosylation of Helicobacter pylori sheathed flagella
Source: PNAS Nexus. 2026 Jan 20;5(2):pgag011. doi: 10.1093/pnasnexus/pgag011 (PMC12880188; doi:10.1093/pnasnexus/pgag011)
Supplement: pgag011_Supplementary_Data [file pgag011_supplementary_data.docx]

**Supplemental Information**

**
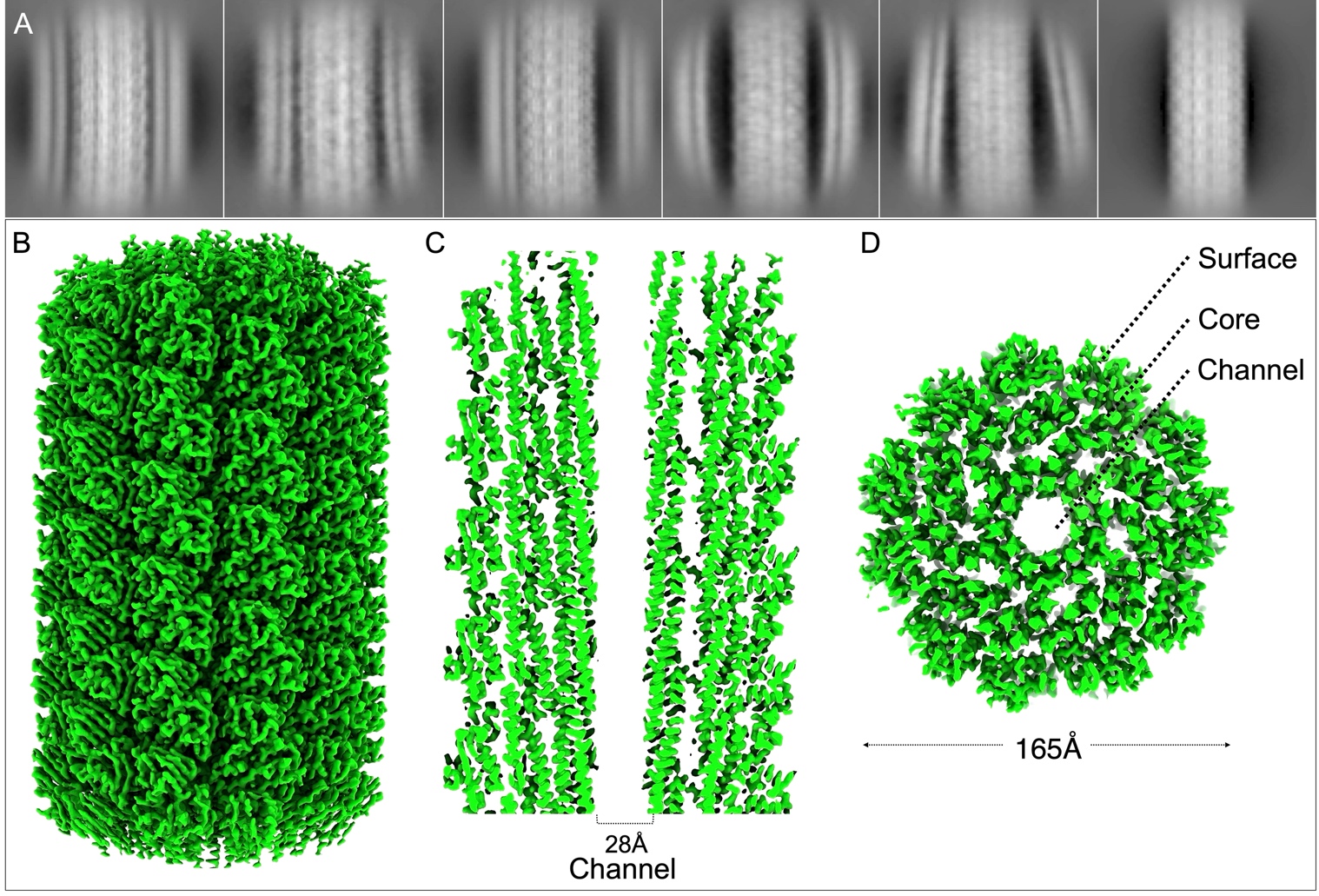
**

**SI Appendix,** **Fig. S1| Cryo-EM reveals the flagellar filament structure enclosed by a membranous sheath with a variable diameter in *H. pylori*.** (**A**) 2D class averages showing the presence of the membranous sheath around the filament. Notably, the spacing between the filament and sheath ranges from 1 to 4nm. No sheath is present in one class. (**B**) 3D surface view of the FlaA filament. (**C**) A vertical cross section of the filament shows filament core and central channel. (**D**) A horizontal cross section of the filament shows the central channel and surface-exposed domains.

**
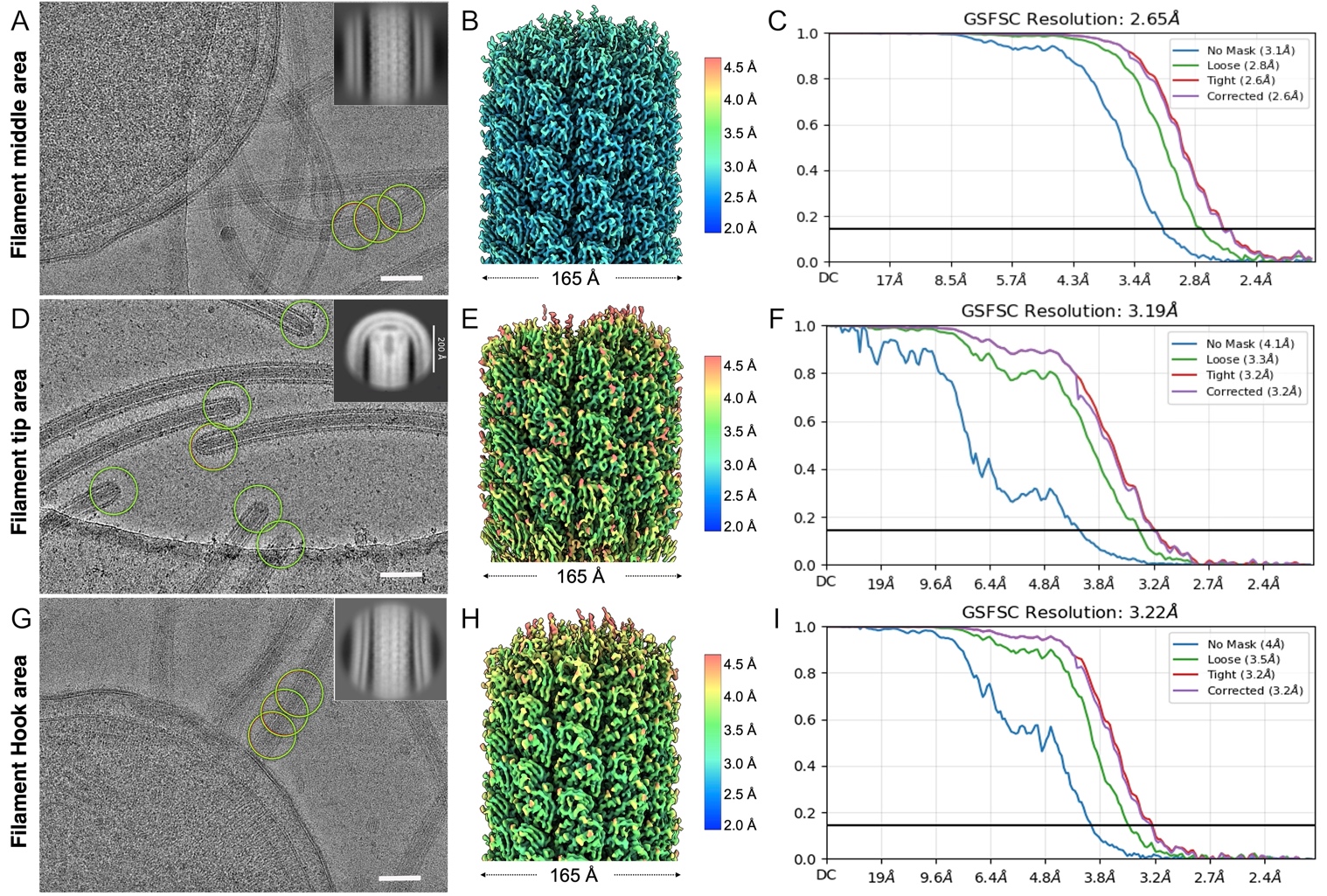
**

**SI Appendix, Fig. S2| Cryo-EM data processing and resolution evaluation.** (**A**, **D**, **G**) Micrographs and selected particles (green circles) of flagellar filaments at the middle (filament tracer auto-picking), tip and near-hook areas (manual picking) with representative 2D classes in inset. scale bars are 50 nm. (**B**, **E**, **H**) Local resolution maps from the middle, tip, and near-hook region, respectively. (**C**, **F**, **I**) Corresponding FSC resolution plots of the structures shown in panels **B**, **E**, and **H**, respectively.

**
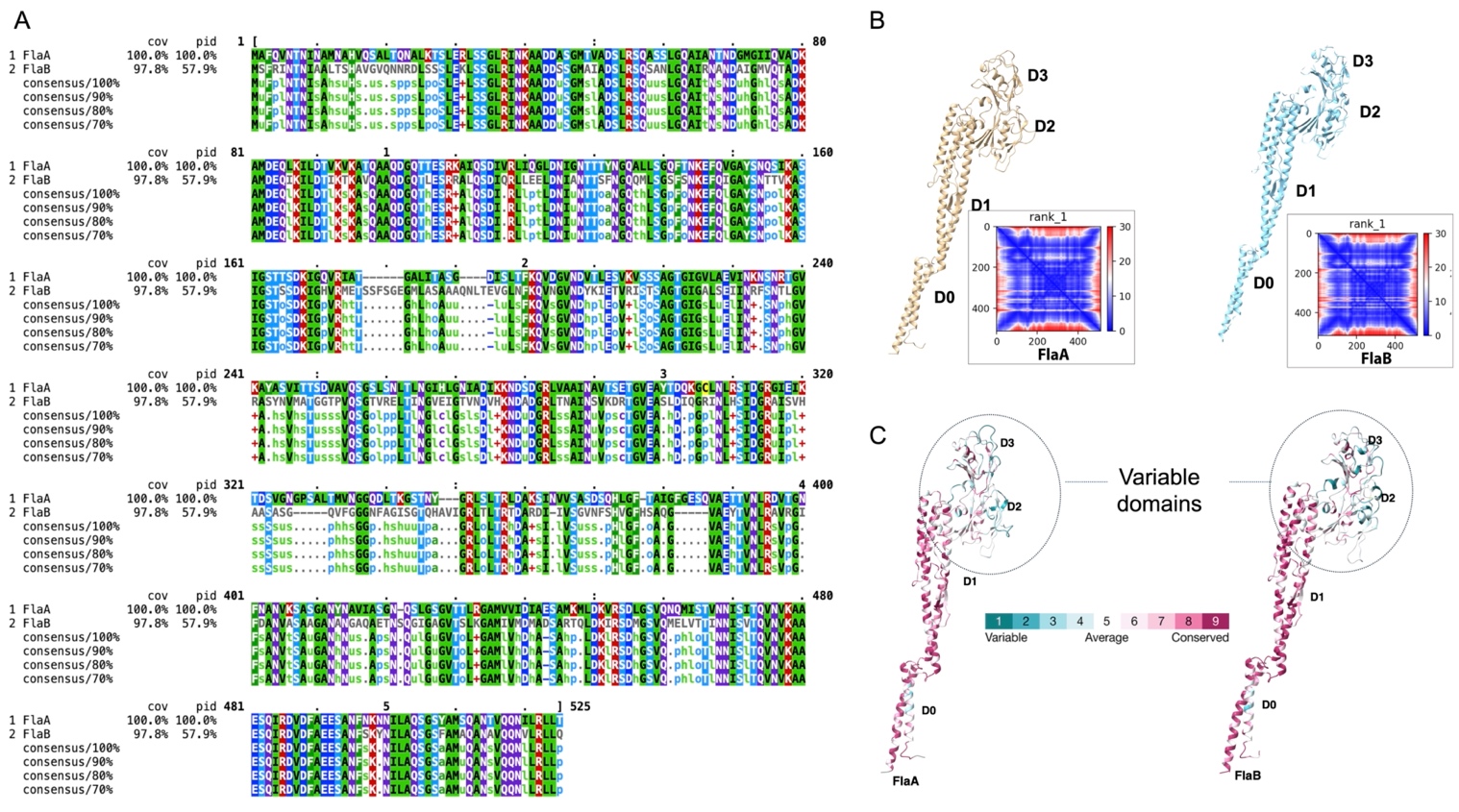
**

**SI Appendix,** **Fig. S3| Flagellin FlaA and FlaB in *H. pylori* are conserved.** (**A**) Sequence alignment of FlaA and FlaB is represented using Mview (49). (**B**) AlphaFold-predicted structures of flagellins FlaA and FlaB with very high confidence (31, 32). (**C**) Analysis of sequence conservative nature of flagellins FlaA and FlaB using Consurf web server (50).

**
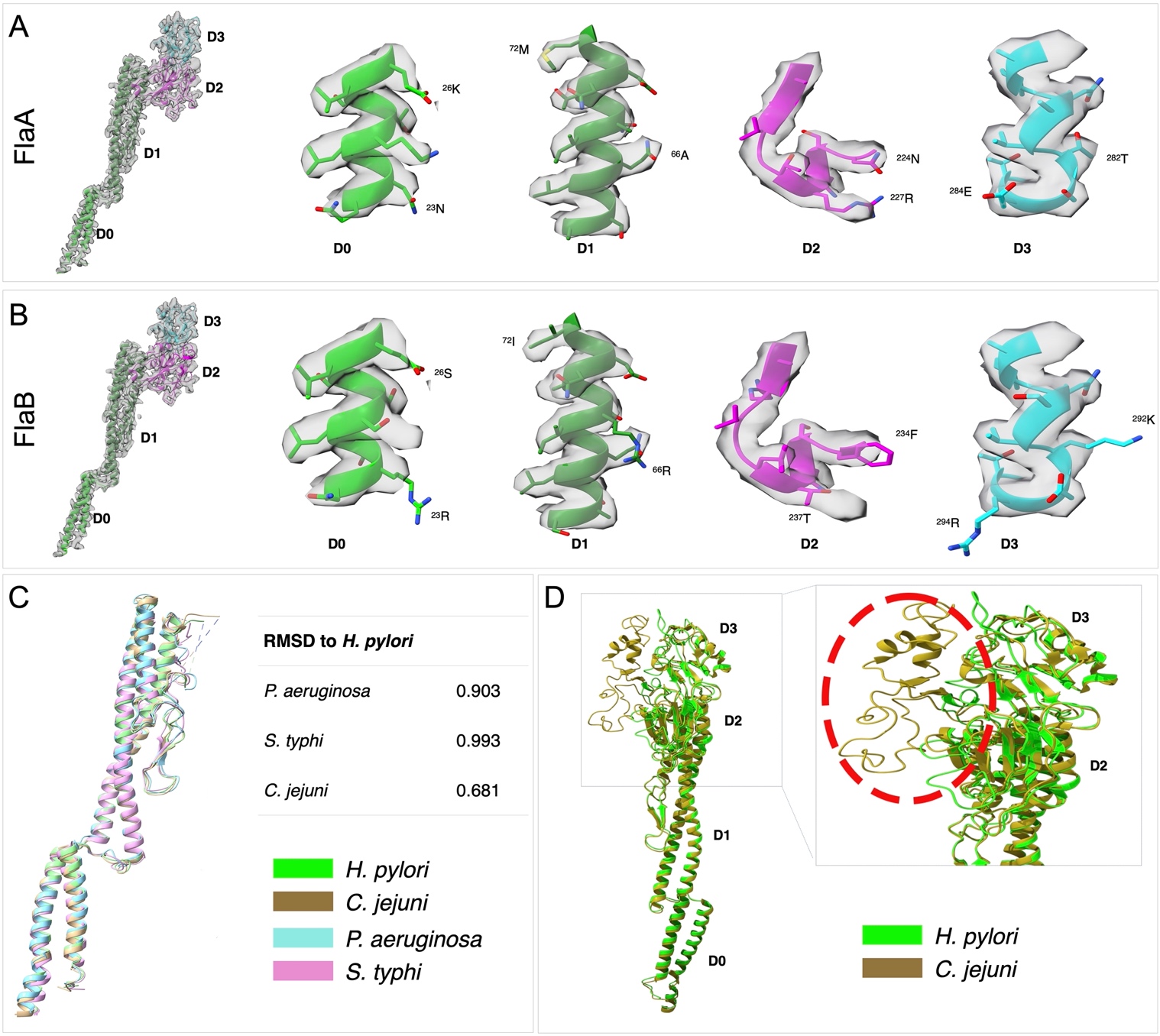
**

**SI Appendix,** **Fig. S4| Comparison of FlaA structure from *H. pylori* with flagellin structures from other bacteria.** (**A**) Left: Ribbon diagram of the refined structure of FlaA fitted into the cryo-EM density map. Right: Zoom-in views of the fitting at different domains of FlaA. (**B**) Left: Ribbon diagram of the refined structure of FlaB fitted into the cryo-EM density map. Right: Zoom-in views of the fitting at different domains of FlaB. Note that some side chains do not fit well into the densities compared to the fitting of FlaA. (**C**) Comparison of D0-D1 domains of *H. pylori* FlaA with those of *P. aeruginosa* FliC, *S. typhi* FliC, and *C. jejuni* FliA. (**D**) Comparison of FlaA structures from *H. pylori* and *C. jejuni*. *C. jejuni* has extra D4 domain which is absent in *H. pylori.*

**
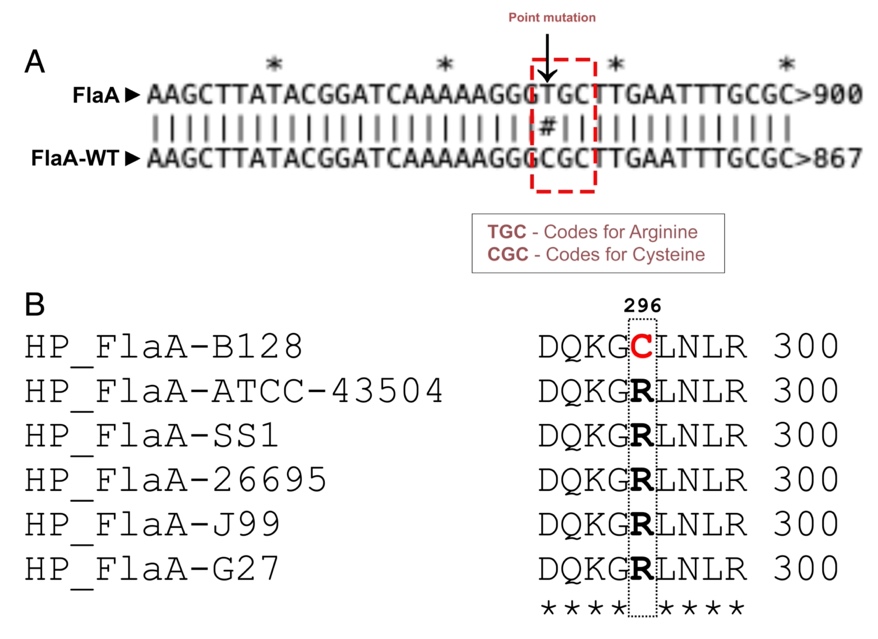
**

**SI Appendix,** **Fig. S5| Sequencing of flagellin FlaA in *H. pylori* B128. (A)** Sequencing confirms the presence of arginine at position 296 in FlaA representing a missense mutation in the *H. pylori* B128 *flaA* sequence in the NCBI and JGI IMG databases*.* (**B)** Image showing sequence alignment of FlaA from *H. pylori* strains- 26695, J99, SS1, G27, and ATCC 43504, indicating presence of arginine residues at 296 position is common in these strains while the wild type *H. pylori* B128 has cysteine at same position. The star represents perfect match of residue.

*
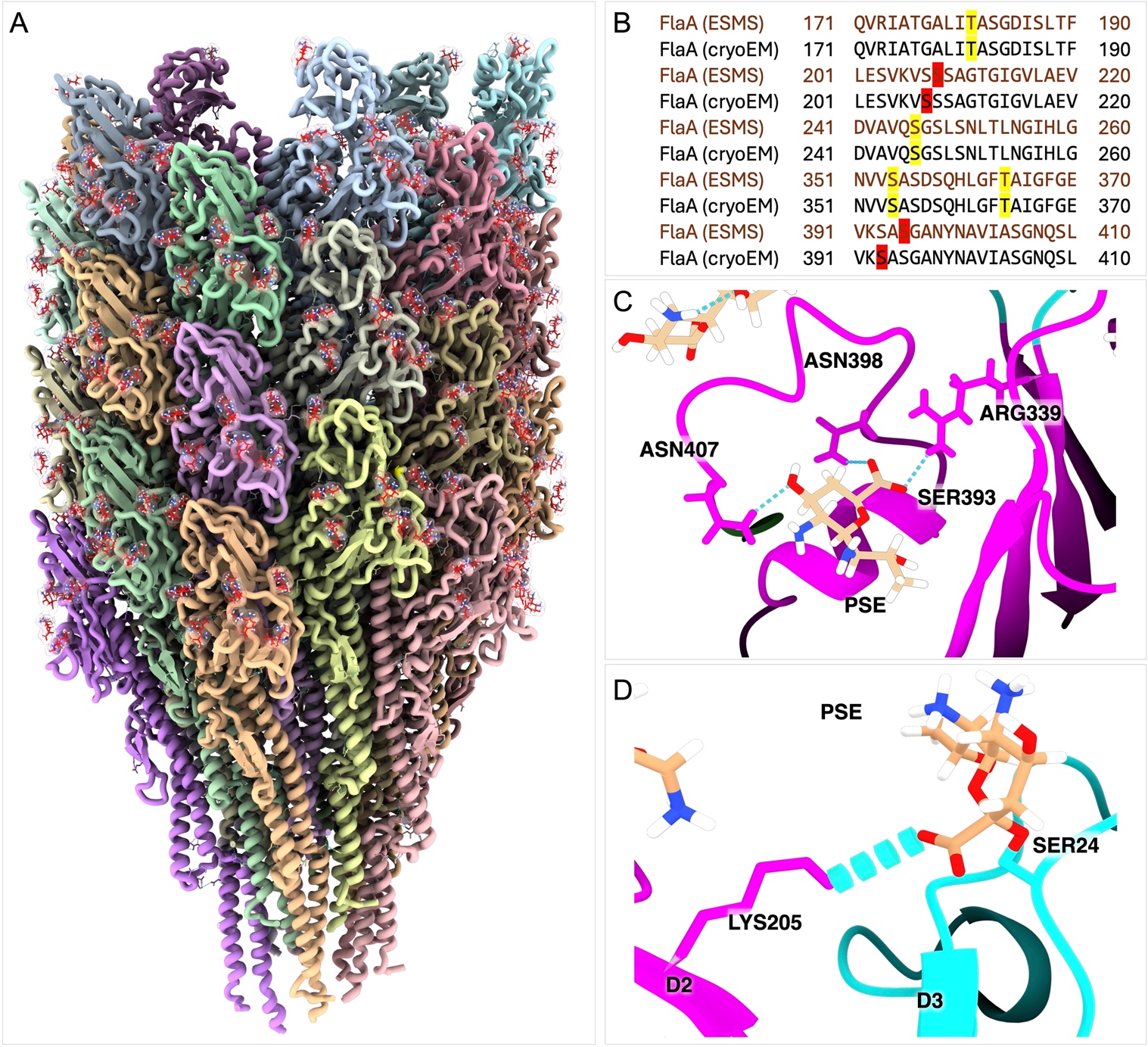
*

**SI Appendix,** **Fig. S6| Glycosylation sites on flagellin FlaA in *H. pylori* B128.** (**A**) The FlaA filament is decorated with extensive glycans. (**B**) Comparison of the glycosylation sites on flagellin FlaA revealed by cryo-EM and those previously identified by ESMS (26). (**C**) Zoomed image of Ser-393 residue participating in glycosylation forms an H-bond with adjacent charged residues in FlaA. (**D**) Pse5Ac7Ac at Ser-246 forms an H-bond with Lys-205 from domain D2 of the adjacent protofilament.

**
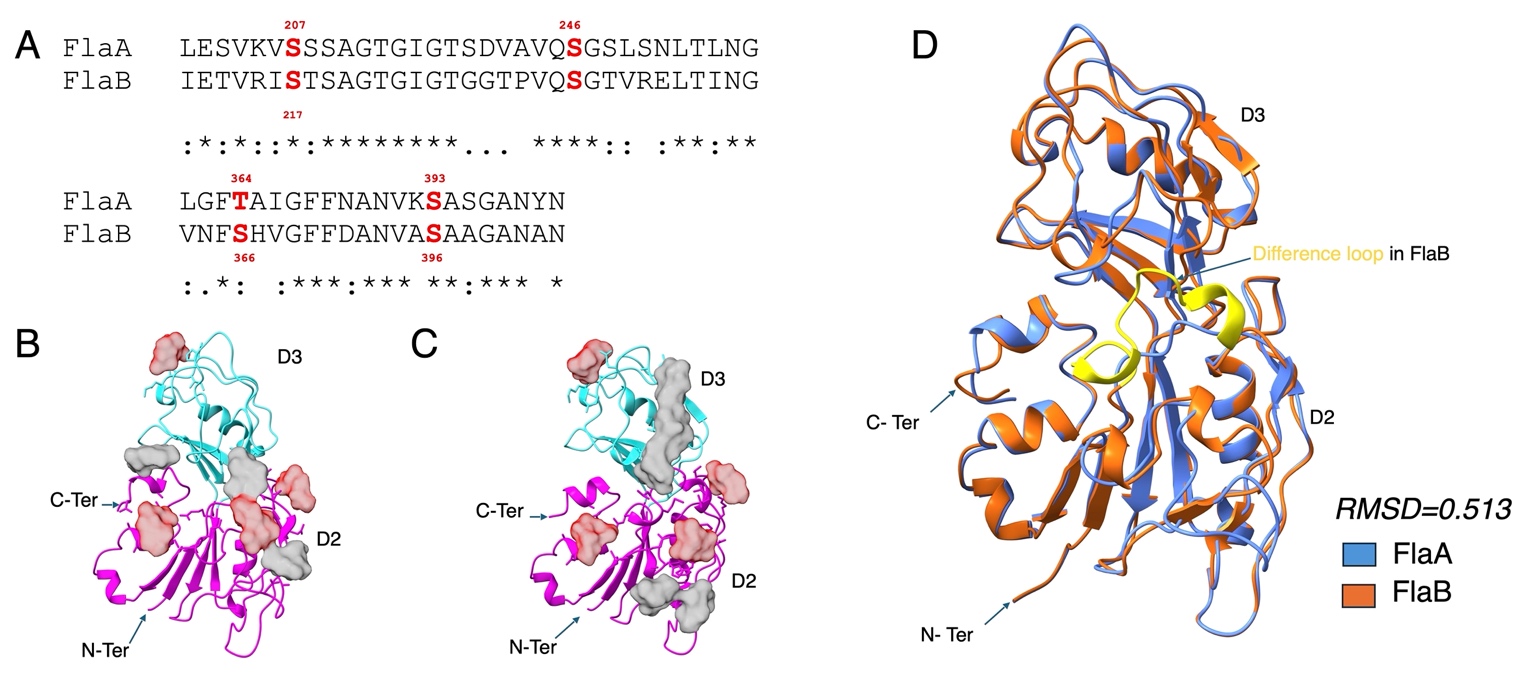
**

**SI Appendix,** **Fig. S7| Comparison of conserved glycosylation sites in FlaA and FlaB.** (**A**) Serine and threonine residues that were identified as being glycosylated in the *in-situ* structures of FlaA and FlaB are indicated in red. Amino acid residues surrounding the O-glycosylated residues that are identical in FlaA and FlaB are indicated with an asterisk (*) and residues with high similarity and lower similarity are indicated with a colon (:) and period (.), respectively. (**B**, **C**) Conserved glycosylation sites (red blob) highlighted in on FlaA and FlaB. Gray color blob represents non conserved glycosylation sites. (**D**) Structure alignment of domain D2 and D3 in FlaA and FlaB. FlaA and FlaB surface exposed domains are structurally similar with RMSD value of 0.513. The major difference between FlaA and FlaB lies in a loop that is present only in FlaB structure (colored yellow).

**
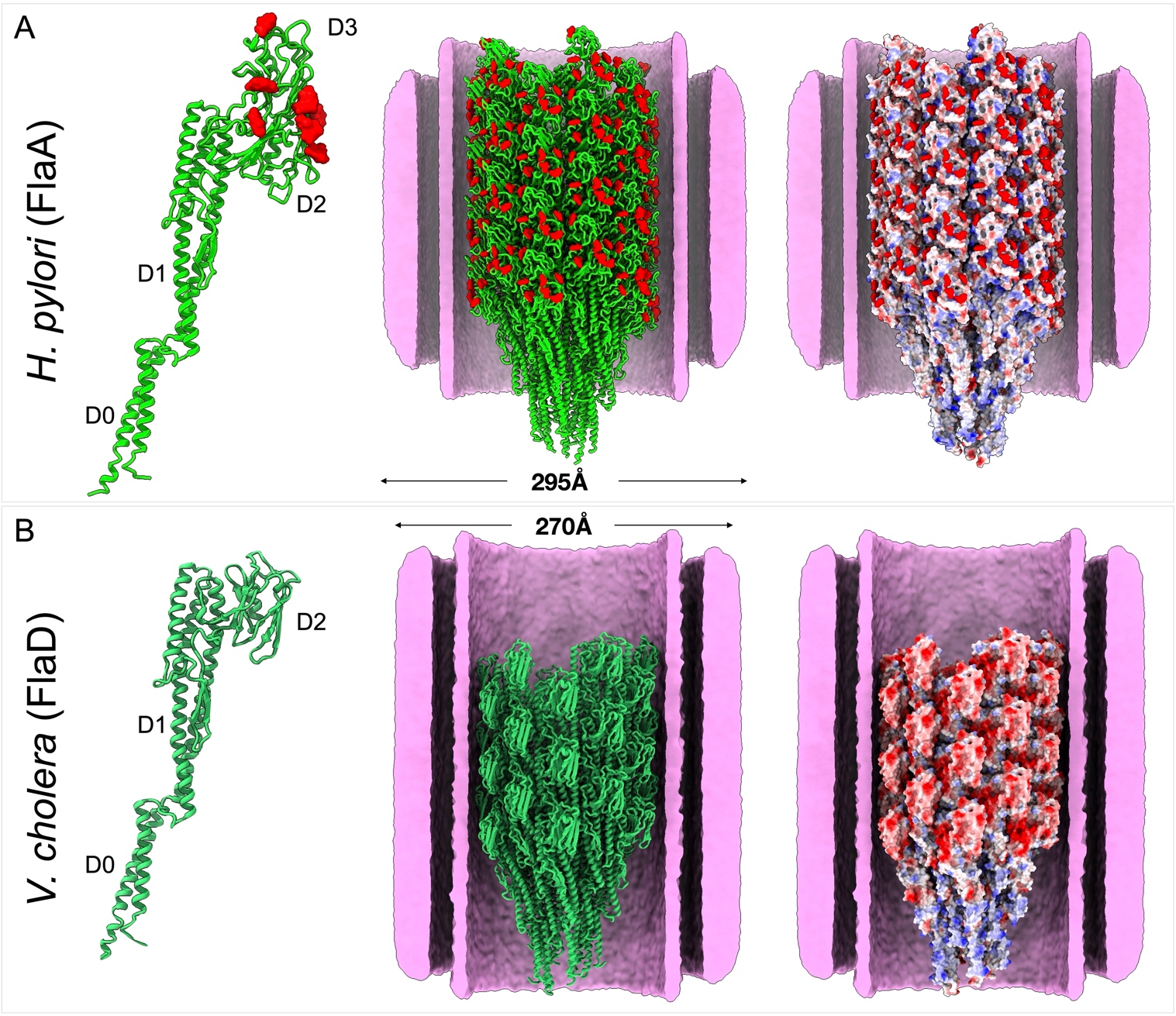
**

**SI Appendix,** **Fig. S8| Comparison of sheathed flagella from *H. pylori* and *V. cholerae*.** (**A**) Left panel: FlaA monomer model of *H. pylori*. Middle panel: FlaA filament model of *H. pylori*. Right panel: Surface charge property of the FlaA filament from *H. pylori.* Pseudaminic acids contribute to the negative charge on the FlaA filament surface in *H. pylori.* (**B**) Left panel: FlaD monomer model of *V. cholerae* (PDB ID: 9N8B). Middle panel: FlaD filament model of *V. cholerae*. Right panel: Surface charge property of the FlaD filament from *V. cholerae* is enriched by the acidic residues on D2 domain.

**SI Appendix,** **Table S1: Cryo-EM data collection, analyses, and structure refinements**

| **Parameters** | **FlaA**  **(middle region)** | **FlaA**  **(Tip region)** | **FlaB**  **(Near-hook region)** |
| --- | --- | --- | --- |
| Magnification | 81000× | | |
| Voltage (kV) | 300 | | |
| Electron exposure (e^-^/Å^2^) | 70 | | |
| Defocus range (um) | -1.0 to -2.0 | | |
| Pixel size (Å) | 1.068 | | |
| **Image processing** | | | |
| Helical rise (Å) | 65.403 | 65.403 | 65.400 |
| Helical twist (°) | 4.682 | 4.682 | 4.680 |
| Final number of particles | 228,410 | 275229 | 60029 |
| Map sharpening B factor (Å^2^) | -57.80 | -39.2 | -26.7 |
| Map resolution (Å) (FSC 0.143) | 2.65 Å | 3.19 Å | 3.22 Å |
| **Refinement and model validation** | | | |
| Model composition | FlaA  (single flagellin) | FlaA  (single flagellin) | FlaB  (single flagellin) |
| Nonhydrogen atoms | 130383 | 84150 | 129228 |
| Number of amino acid residues | 16764 | 11176 | 16929 |
| Number of ligands | 231 | 154 | 297 |
| B factors (Å^2^)  Protein  Ligand | 33.01/208.03/76.04  72.34/148.88/103.67 | 37.62/215.28/88.26  57.27/167.72/100.42 | 54.81/271.39/112.03  103.24/217.61/157.34 |
| Root-mean-square deviations  Bond lengths (Å)  Bond angles (deg) | 0.005 (0)  0.480 (132) | 0.003 (0)  0.398 (35) | 0.002 (0)  0.450 (13) |
| MolProbity score | 2.14 | 2.74 | 2.45 |
| Clashscore | 20.14 | 32.64 | 39.28 |
| Rotamer outliers (%) | 1.53 | 3.47 | 1.86 |
| Ramachandran favored (%) | 96.79 | 95.28 | 96.99 |
| Ramachandran allowed (%) | 3.01 | 4.70 | 3.01 |
| Ramachandran outliers (%) | 0.20 | 0.02 | 0.01 |
| PDB ID | 9YGU | Not deposited | 9YH1 |
| EMDB ID | EMD-72941 | Not deposited | EMD-72948 |
